# miR-183 promotes radioresistance of lung adenocarcinoma H1299 cells via epithelial-mesenchymal transition

**DOI:** 10.1590/1414-431X20209700

**Published:** 2021-04-02

**Authors:** Yi Huang, Mengmei Zhang, Yang Li, Jihang Luo, Yuanyan Wang, Wenjing Geng, Ze Yang, Hu Ma, Yuju Bai

**Affiliations:** 1 Department of Oncology, The Second Affiliated Hospital of Zunyi Medical University, Zunyi Medical University, Gui Zhou, Zun Yi, China; 2 Zunyi Medical and Pharmaceutical College, Gui Zhou, Zun Yi, China

**Keywords:** Lung adenocarcinoma, miRNA-183, ZEB1, EMT, Radioresistance

## Abstract

Lung adenocarcinomas are usually sensitive to radiation therapy, but some develop resistance. Radiation resistance can lead to poor patient prognosis. Studies have shown that lung adenocarcinoma cells (H1299 cells) can develop radioresistance through epithelial-mesenchymal transition (EMT), and this process is regulated by miRNAs. However, it is unclear which miRNAs are involved in the process of EMT. In our present study, we found that miR-183 expression was increased in a radioresistant lung adenocarcinoma cell line (H1299R cells). We then explored the regulatory mechanism of miR-183 and found that it may be involved in the regulation of zinc finger E-box-binding homeobox 1 (ZEB1) expression and mediate EMT in lung adenocarcinoma cells. qPCR results showed that miR-183, ZEB1, and vimentin were highly expressed in H1299R cells, whereas no difference was observed in E-cadherin expression. Western blot results showed that ZEB1 and vimentin were highly expressed in H1299R cells, while E-cadherin expression was decreased. When miR-183 expression was inhibited in H1299R cells, radiation resistance, proliferation, and cell migration were decreased. The expression of ZEB1 and vimentin in H1299R cells was decreased, while the expression of E-cadherin was increased. Moreover, miR-183 overexpression in H1299 cells enhanced radiation resistance, proliferative capacity, and cell migration ability. The expression of ZEB1 and vimentin in H1299 cells was increased, while that of E-cadherin was decreased. In conclusion, miR-183 may promote EMT and radioresistance in H1299 cells, and targeting the miR-183-ZEB1 signaling pathway may be a promising approach for lung cancer treatment.

## Introduction

Radiation therapy is considered one of the main treatment strategies for lung adenocarcinoma. However, during radiotherapy, some tumor cells are tolerant to radiation, reducing the efficacy of radiotherapy (1-3). Therefore, evaluation of the molecular mechanisms of radiotherapy resistance and identification of ways to increase the radiosensitivity of tumors are of great significance for improving the efficacy of tumor therapy.

Ideally, after X-ray exposure, tumor cells undergo DNA damage and activate programmed cell death and mitotic disruption pathways, eventually leading to death (4). However, during DNA damage, epithelial-mesenchymal transition (EMT) of tumor cells during radiation promotes anti-radiation effects (5,6). Recent studies on radioresistance have shown that many radioresistant tumor cells undergo phenotypic changes during EMT (7). In addition, the cytokines and transcription factors associated with EMT directly participate in the occurrence of radioresistance in tumor cells (8,9). Moreover, tumor cells possess stem cell-like characteristics after EMT, leading to a certain resistance to radiotherapy (5,10).

MicroRNAs (miRNAs) are posttranscriptional regulators that play an important regulatory role in the biological characteristics of tumors. miR-183 is associated with the occurrence, development, invasion, metastasis, and drug resistance of various human solid tumors. According to previous studies, miR-183 is overexpressed in lung cancer cells, which in turn promotes their migration and invasion (11,12). In a previous experimental study, H1299R cells changed from having an original short spindle shape to having a long spindle shape, accompanied by pseudopod formation. The cells also lost the epithelial cell morphology that was characterized by tightly connected polygons and instead became loosely connected, similar to fibroblasts (13). It has been suggested that H1299 cells undergo EMT when continuously irradiated with X-rays (Supplementary Figure S1A and B). We used quantitative polymerase chain reaction (qPCR) to detect the differences in miRNA expression between H1299 cells and H1299R cells. The results revealed that miR-183 was highly expressed in H1299R cells, suggesting that miR-183 played an important role in the maintenance of radioresistance in lung adenocarcinoma H1299 cells. We hypothesized that miR-183 might mediate EMT processes, leading to radioresistance in H1299R cells exposed to continuous X-rays. Thus, we explored the relationship between miR-183 and EMT and their effects on radiosensitivity through experimental methods, such as western blot, CCK-8 assay, clonogenic assay, and qPCR.

## Material and Methods

### Cell culture, proliferation, cryopreservation, and acquisition of H1299R cells

Human lung adenocarcinoma H1299 cells were obtained from the CAS Shanghai Life Science Cell Bank (China).

We used monolayer culture for H1299 cells and radioresistant H1299R cells and cultured the cells using conventional methods in medium that contained 10% fetal calf serum and double-antibody RPMI 1640 at 37°C in 5% CO_2_ and saturated humidity. The cells were used when confluency reached 85-90%, the cells grew well, with no contamination and no suspicion of diseased cells. We then continued to cultivate the next generation or chose to cryopreserve the cells as needed.

The H1299R cells were obtained after continuous exposure of H1299 cells to X-rays, (irradiation conditions: 6 MV X-rays produced by a linear accelerator, an irradiation field of 10×10 cm, and a dose rate of 400 cGy/min 100 cm from the source). H1299R cells stably proliferated for 10 generations (1 month) or more. It was necessary to maintain irradiation when H1299R cells were routinely cultured beyond 3 months and proliferated for more than 15 generations. The irradiation dose was maintained at 30 Gy until irradiation was complete, and the method was the same as the conventional division method for the induction of radioresistant cells. After the final irradiation and cell proliferation had been stably maintained for 10 generations (1 month), experiments related to the radioresistant cells were conducted.

### CCK-8 assay

H1299, H1299R, H1299R-shRNA-NC, H1299R-shRNA-miR183, H1299-EGFP-NC, and H1299-EGFP-miR183 cells were diluted to 20 cells/µL and inoculated into 96-well plates. Then, 200 µL of serum-containing medium was added to each well, with 5 sets of wells for each group of cells, and a group consisting of medium alone was used as a blank group for routine culture. Seven sets of 96-well plates were configured according to the test requirements for 1-7 consecutive days after separate irradiation. The 96-well plates of each group of cells were inoculated with a sublethal dose of 6 Gy of irradiation. The absorbance value of each well was measured at 450 nm by a microplate reader (Bio-Rad, USA). The cell growth curve of each group after culturing for 1-7 days was obtained by the following formula: absorbance value of the cells = the absorbance value of each well - the absorbance mean of the blank control group.

### Clonogenic assay

The experimental cells were treated with a dose gradient of 0, 2, 4, 6, and 8 Gy with 200, 400, 800, 1600, and 3200 cells inoculated in each well, respectively. Stop culture was performed when macroscopic colony formation occurred on the culture plate. After fixing the cells with methanol, Giemsa (Soleil Bao Company, China) staining was used to stain the cells. Finally, the colony formation rate was calculated using the following formula: clonal formation rate = (number of clones / number of cells inoculated) × 100%.

### Wound healing assay

After transferring H1299 cells and culturing them for 24 h, a straight wound was created using a 200-µL sterile tip in the middle of the well. The cells were washed with PBS twice to smooth the edges of the scratch and remove floating cells. After incubation (37°C, 5% CO_2_) for 0, 6, 12, and 24 h, images of the migrating cells were observed under an optical inverted microscope (Olympus Corp., Japan). The experiments were repeated 3 times.

### qPCR

The primers were designed by Shanghai Shenggong Bioengineering Co., Ltd. (China) and the sequences are reported in Table 1. Total cellular RNA was extracted by using TRIzol reagent (Invitrogen Corporation, USA). Reverse transcription reactions of miR-183, U6, ZEB1, and EMT-related molecular markers were conducted. The 2^-ΔΔct^ formula was used to calculate the relative mRNA expression levels (14). U6 and β-actin expression were used for normalization.

**Table 1:** qPCR primers and sequences.

Primer name	Primer sequence (5′-3′)
RT Primer of Has-miR-183-5p	GTCGTATCCAGTGCAGGGTCCGAGGTATTCGCACTGGATACGACCAGTGAAT
Forward primer of Has-miR-183-5p	GCGGCGGTATGGCACTGGTAGA
Reverse primer of Has-miR-183-5p	GCGGGTGCAGGGTCCGAGGT
RT Primer of Homo U6	AACGCTTCACGAATTTGCGT
Forward primer of Homo U6	CTCGCTTCGGCAGCACA
Reverse primer of Homo U6	AACGCTTCACGAATTTGCGT
Forward primer of Homo ZEB1	GCTTGTGATTTGTGTGACAAGA
Reverse primer of Homo ZEB1	AATCGCATGTGTTCAATCAA
Forward primer of Homo E-cadherin	GAGTGCCAACTGGACCATTC
Reverse primer of Homo E-cadherin	ACCCACCTCTAAGGCCATCT
Forward primer of Homo Vimentin	AGATGGCCCTTGACATTGAG
Reverse primer of Homo Vimentin	CCAGAGGGAGTGAATCCAGA
Forward primer of Homo β-actin	CCTCGCCTTTGCCGATCC
Reverse primer of Homo β-actin	GGGCACGAAGGCTCATCATT

### Western blot

Cell lysis was performed with PMSF (phenylmethylsulfonyl fluoride) lysis buffer (Soleil Bao Company). A BCA Protein Assay Kit (Soleil Bao Company, China) was used to determine the protein concentration. Electrophoretic blotting was performed to transfer the proteins from the gel to a nitrocellulose membrane. All the primary antibodies were incubated with blotting at 4°C overnight. β-actin was used as an internal reference protein. ImageJ v1.8.0 software (NIH, USA) was used to calculate the expression level of the relevant protein bands, and the numerical values are reported as means±SD. All the primary antibodies were purchased from Abcam, (USA) including vimentin (ab92547) (diluted at 1:5000), E-cadherin (ab40772) (diluted at 1:5000), and ZEB1 (ab203829) (diluted at 1:500). The secondary antibodies goat anti-rabbit IgG (H L) and mouse/human ads-HRP were purchased from Southern Biotech (No. 4050-05) (diluted at 1:20000). HRP-labeled β-actin, from Shanghai Kangcheng Biological (No. KC-5A08) (diluted at 1:10000), was used as the internal reference antibody. Medical X-ray film from was purchased from Kodak Company (USA), and Immobilon Western chemiluminescent HRP substrate and Immobilon-P transfer membranes were purchased from Millipore (Germany).

### Lentiviral vector infection cell experiment

The overexpression/silencing lentiviral vector and negative control virus were constructed and packaged by Shanghai Genechem Co. Ltd., (China) and the virus itself carried the enhanced green fluorescent protein (EGFP) gene. The appropriate amount of virus was calculated based on the MOI (multiplicity of infection) value and the cell number. The concentrated lentivirus was transfected (MOI=2) into human lung adenocarcinoma cells and irradiated H1299R cells. After transfection for 72 h, an inverted fluorescence microscope (Nikon Corp, Japan) was used for observation: no viable expression was observed in the blank control group without virus transfection; green fluorescence was observed in the negative control group and the silenced miR-183 expression group. After 72 h of transfection, the expression of the lentiviral reporter gene EGFP was observed. If the fluorescence rate was greater than 80%, then the infection was considered successful. H1299R cells transfected with negative control lentivirus were named H1299R-shRNA-NC, and H1299R-shRNA-miR183 cells transfected with miR-183 that interfered with the expression of lentivirus were named H1299R-shRNA-miR183. After the cells were cultured for a period of time, qPCR experiments were conducted to confirm the downregulation of miR-183 expression. The overexpression/silencing of the lentiviral vector and sequences are presented in Table 2.

**Table 2:** The overexpression/silencing of lentiviral vector and sequences.

	No.	STEM
Silencing of lentiviral vector	GV280	hU6-MCS-Ubiquitin-EGFP-IRES-puromycin
Insertion sequence of silencing lentiviral vector virus	hsa-miR-183-5p-inhibition(18159-1)	TATGGCACTGGTAGAATTCACT
Insertion of silencing lentiviral vector negative control virus	CON137	TTCTCCGAACGTGTCACGT
Overexpression of lentiviral vector	GV369	Ubi-MCS-SV40-EGFP-IRES-puromycin
Insertion sequence of overexpression lentiviral vector virus	hsa-mir-183(17525-1)	CCAAGGGAGTGGGCAGGCTAGGAGCAGGGAACGGGCATCGTGGGCCGCTGGTCTCTCCGCAGGGTCGGCAGGCCGCAGAGTGTGACTCCTGTTCTGTGTATGGCACTGGTAGAATTCACTGTGAACAGTCTCAGTCAGTGAATTACCGAAGGGCCATAAACAGAGCAGAGACAGATCCACGAGGGCCTCCGGAGCACCTTACCCACTTCTGCCTTGAGTGCTCCTAGACGTCGGAAACAGGCTGCTTCCAAGGGTGCAGGGA
Insertion sequence of overexpression lentiviral vector negative control virus	CON238	NA

### Statistical analysis

SPSS 20.0 statistical software (IBM, USA) was used for data processing. The measurement data are reported as means±SD. Comparison of the means was performed by the *t*-test. The test level was α=0.05, and the difference was statistically significant at P<0.05.

## Results

### Radioresistance in H1299R cells compared to that in H1299 cells

**Figure 1:**
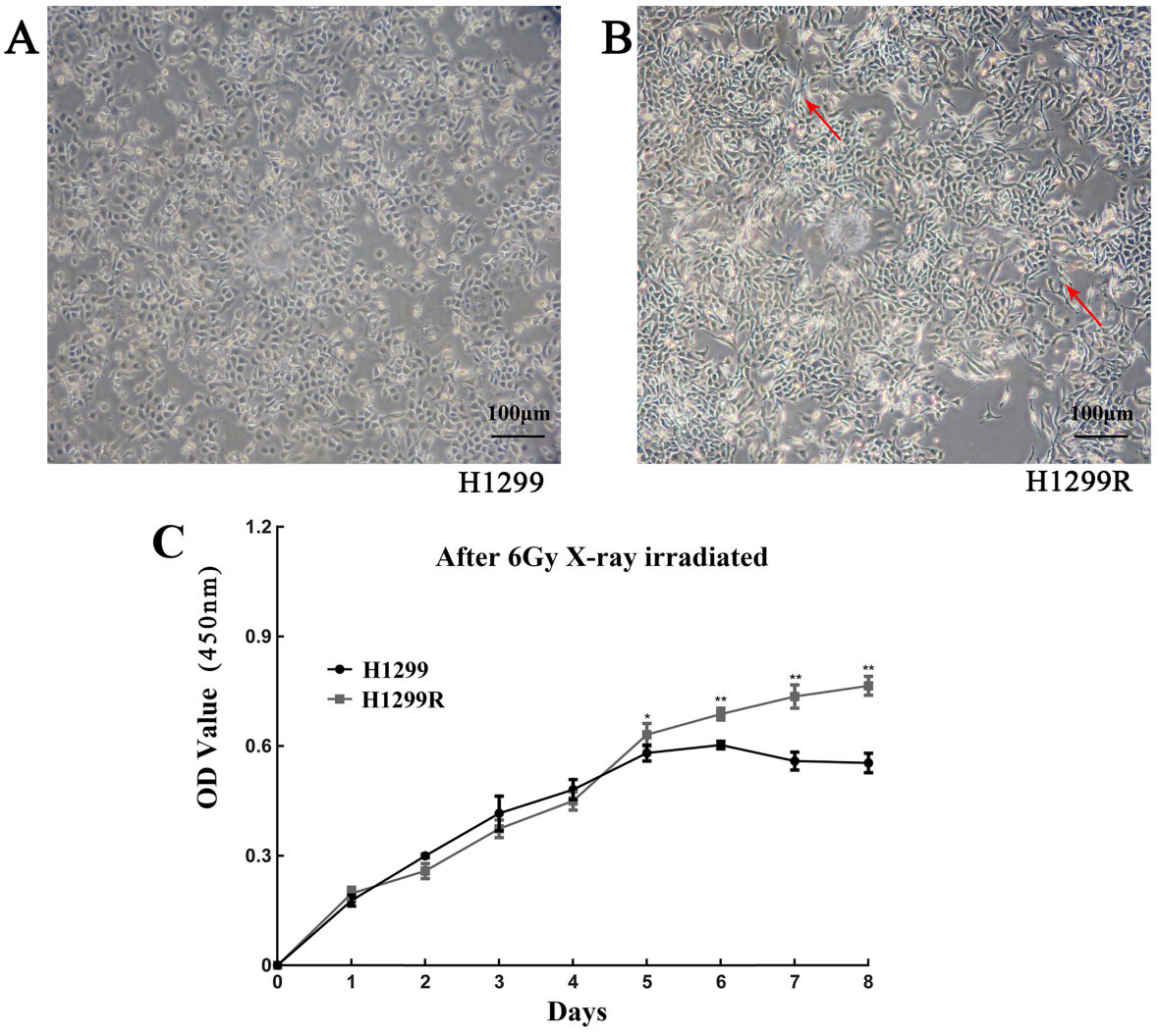
Morphological observation of H1299 (**A**) and H1299R (**B**) cells (magnification 20×; scale bar: 100 μm). Red arrows indicate pseudopod formation. **C**, Changes in the proliferative capacity of H1299 and H1299R cells. Data are reported as means±SD (n=3). *P<0.05, **P<0.01 (*t*-test).

After continuous X-ray irradiation, the morphology of lung adenocarcinoma cells was changed. Compared with parental H1299 cells, H1299R cells showed a long fusiform shape with pseudopod formation, and the original epithelial cell morphology with polygonal tight junctions was also lost (Figure 1A and B). The CCK-8 assay showed that the proliferation of the H1299R cells was significantly higher than that of the parental cells on days 5-7 (all P<0.05) (Figure 1C). This result indicated that H1299R cells became resistant to X-rays by promoting proliferation. The colony formation experiments showed that, at the same irradiation dose, the number of colonies formed by the parental cells was significantly reduced compared to that formed by the H1299R cells, and the resistant cells had a stronger ability to be cloned, causing a certain level of resistance to radiation compared with H1299R cells. The multitarget single-hit model was used to fit the survival scores of cells with different metrologies and calculate the radiosensitivity parameters of each group. The results showed that the D0, Dq, and SF2 values of the H1299R cells were significantly increased compared with those of the H1299 cells (Table 3). These results indicated that H1299R cells have stronger colony-forming abilities than H1299 cells as well as enhanced radiological resistance.

**Table 3:** Radiosensitivity parameters.

Cells	D_0_	D_q_	N	SF_2_	SER
H1299R	3.018412	0.1690311	1.056	0.501294312±0.168487766*	1
H1299R-shRNA-NC	2.97619	-0.3464286	0.8836	0.476918775±0.0075201010	1.014186594
H1299R-shRNA-miR183	2.255809	-0.8996165	0.6012	0.271951284±0.0101135099*	1.338061866
H1299	2.747252747	-1.189285714	0.5671	0.309003681±0.0309269512	1
H1299-EGFP-NC	2.760143527	-1.337013525	0.5156	0.301589242±0.168818422	0.99532967
H1299-EGFP-miR183	2.801905296	-0.16391146	0.9415	0.474246149±0.0266155100*	0.980494505

H1299R-shRNA-NC *vs* H1299R-shRNA-miR183,*P<0.05, n=3; H1299-EGFP-NC *vs* H1299-EGFP-miR183, *P<0.05, n=3; H1299 *vs* H1299R *P<0.05, n=3. D_0_: mean lethal dose; Dq: quasi-threshold dose, which is the intersection of straight line portion of the curve and dose axis intersecting the 100% survival level, the size of the shoulder area, and the smaller value represents the sub-lethal repair ability; N: the number of extrapolation, which is the focal point that intersects the survival axis by straight line portion of the curve, and reflects the number of radiosensitive areas contained in the cells; SF2: surviving fraction at 2Gy; SER: sensitization enhancement ratio.

### H1299R cells expressed high levels of miR-183 and low levels of E-cadherin

**Figure 2:**
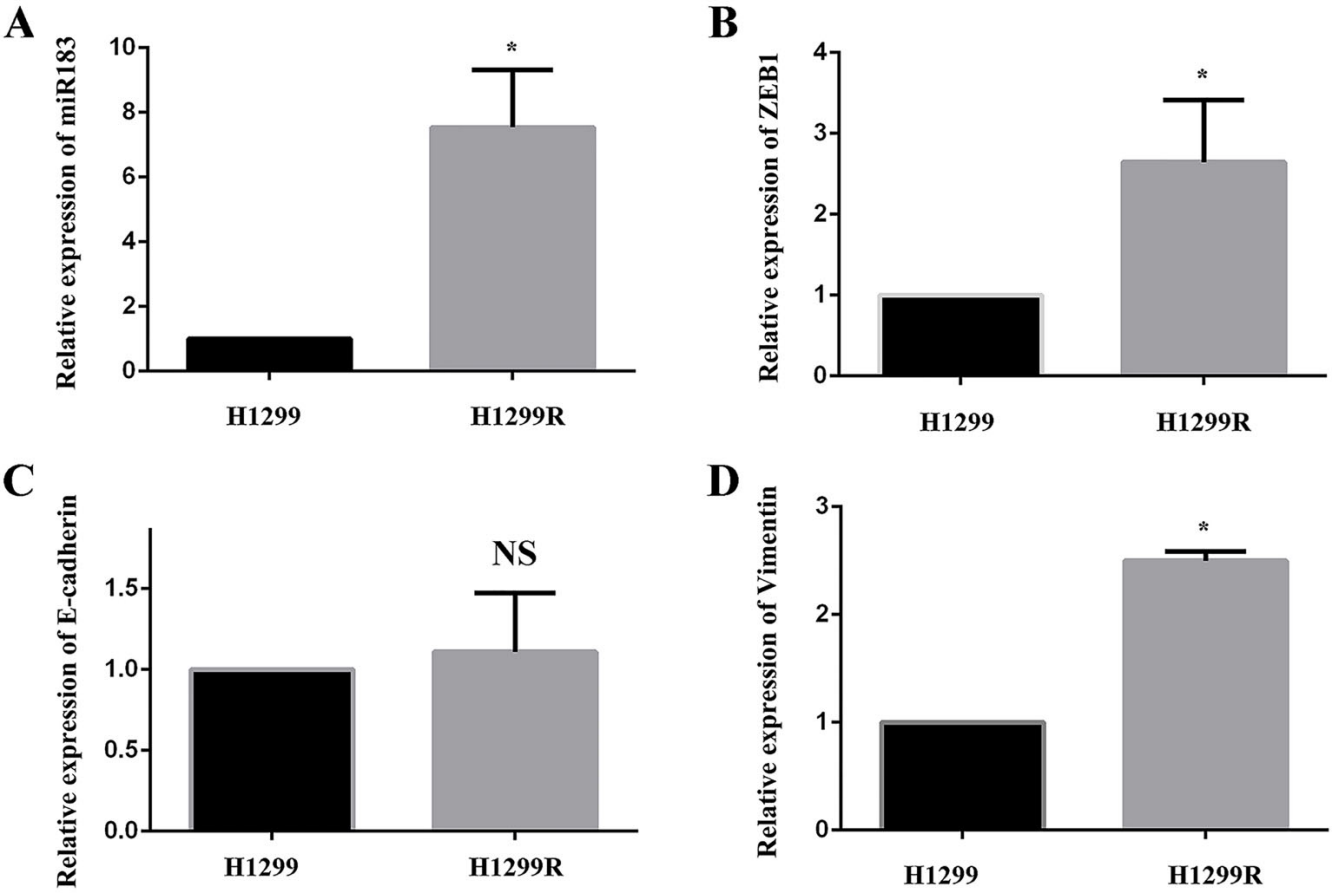
**A-D**, mRNA expression levels of miR-183, ZEB1, E-cadherin, and vimentin in H1299 and H1299R cells. Data are reported as means±SD (n=3). *P<0.05; NS, P>0.05 (*t*-test).

**Figure 3:**
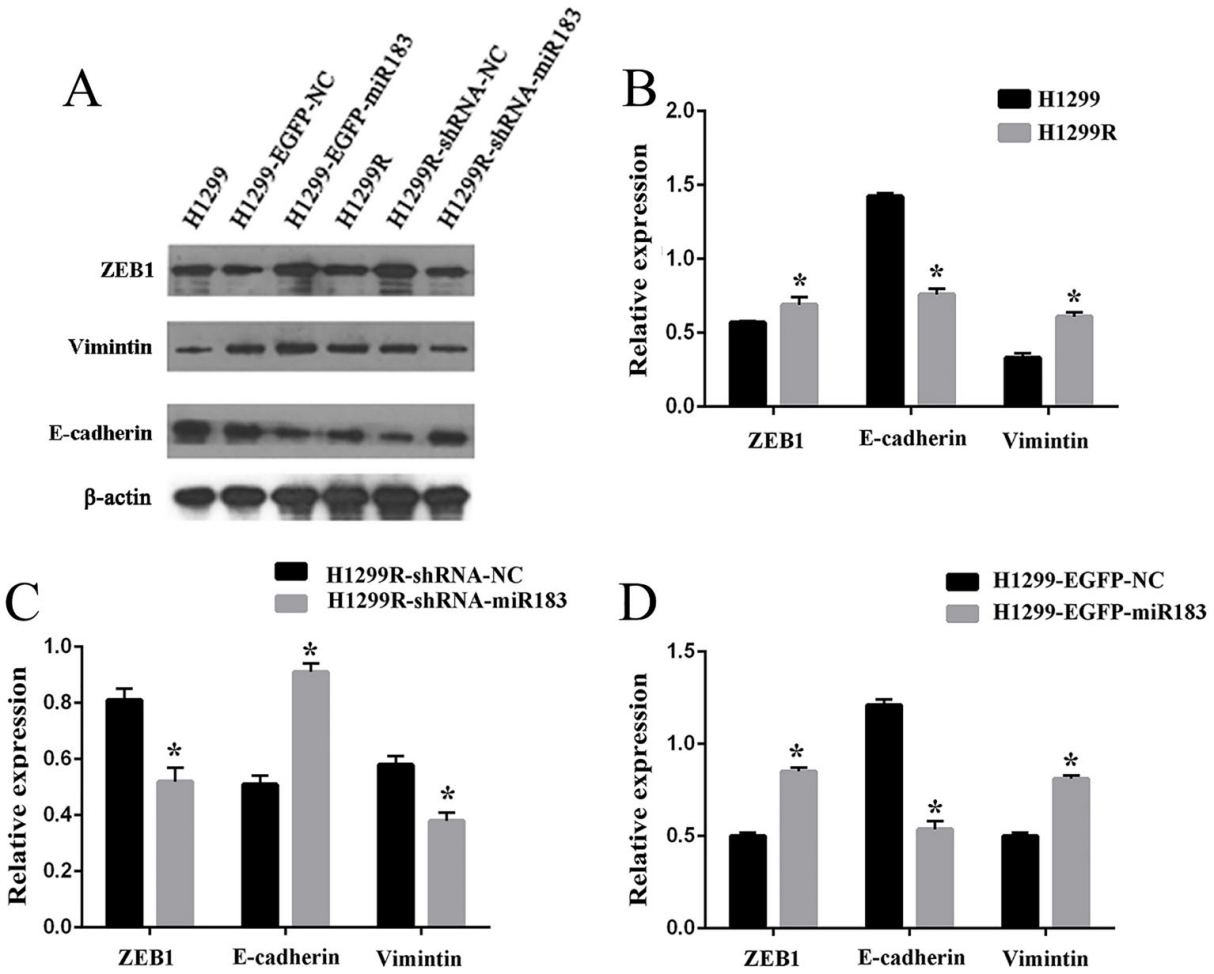
**A** and **B**, Protein expression of ZEB1, E-cadherin, and vimentin in H1299, H1299-EGFP-NC, H1299-EGFP-miR183, H1299R, H1299R-shRNA-NC, and H1299R-shRNA-miR183 cells, **C**, in the H1299R negative control (NC) group and H1299R miR-183-downregulated expression group, and **D**, in the H1299 negative control group and H1299 miR-183-overexpression group. Data are reported as means±SD (n=3). *P<0.05 (*t*-test).

In previous experiments (13), we found that the expression of miR-183 was increased in H1299R cells. When searching for miR-183 target genes by bioinformatics, we found that miR-183 can directly act on the 3′-UTR of ZEB1. In addition, through gene ontology (GO) analysis of the target gene enrichment entries of miR-183, we found that ZEB1 was enriched in the two most important GO entries. These results suggested that miR-183 may initiate the process of EMT by regulating the expression of ZEB1 (Supplementary Figure S1). Next, we sought to examine the potential mechanism that was involved in the radioresistance of H1299R cells. Real-time PCR showed that the miR-183, ZEB1, and vimentin levels were significantly higher in H1299R radioresistant cells than in the parental H1299 cells (all P<0.05), but the expression of E-cadherin was comparable between these cell types (P>0.05) (Figure 2A-D). Moreover, western blotting showed that the expression of ZEB1 and vimentin was high in H1299R cells (all P<0.05), while the expression of E-cadherin was decreased (P<0.05) (Figure 3A and B). When miR-183 was down-regulated, the expression of ZEB1 and vimentin decreased, while the expression of E-cadherin increased, and when miR-183 was up-regulated, the protein expression was opposite to the previous one (P<0.05) (Figure 3C and D). These findings suggested that miR-183 may increase the radioresistance of H1299R cells by promoting the development of EMT.

### miR-183 promoted radiation resistance by activating the EMT signaling pathway

**Figure 4:**
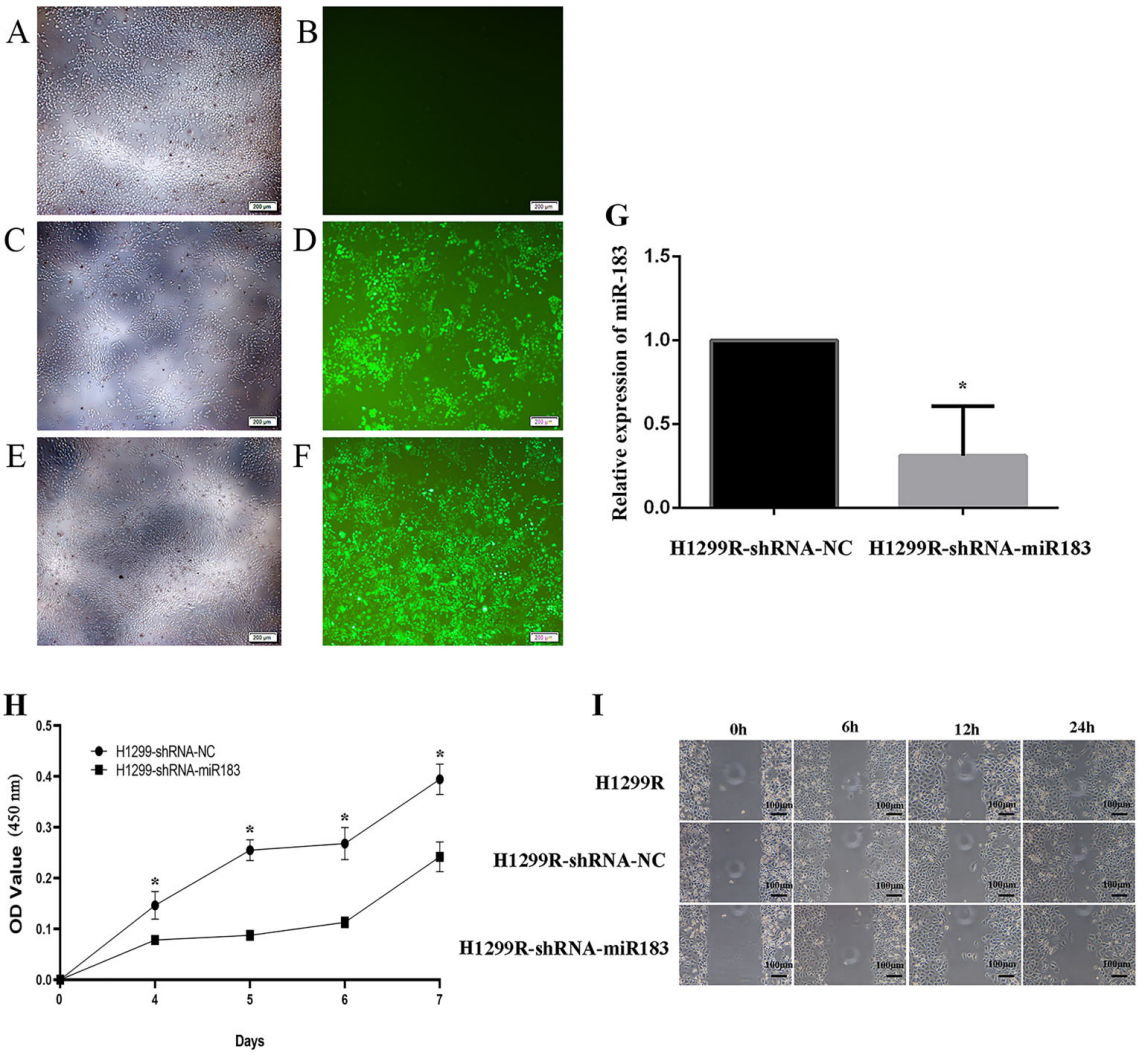
**A-F**, Expression of EGFP under a fluorescence microscope after H1299R cells were infected with lentivirus. **A** and **B**, H1299R blank control group; **C** and **D**, H1299R negative control group; **E** and **F**, H1299R miR-183-silenced expression group (scale bar: 200 μm). **G**, Expression of miR-183 was detected by qPCR after transfection of H1299R cells with lentivirus (H1299R-shRNA-miR183 *vs* H1299R-shRNA-NC. **H**, Effect on cell proliferation after H1299R cells were infected with virus (H1299R-shRNA-NC: H1299R negative control group; H1299R-shRNA-miR183: H1299R miR-183-low expression group). Data are reported as means±SD (n=3). *P<0.05 (*t*-test). **I**: Scratch healing test after H1299R cells were infected with virus (scale bar: 100 μm).

To study the role of miR-183 in the radioresistance of lung adenocarcinoma cells, a lentiviral transfection experiment was performed by transfecting concentrated lentivirus into H1299R cells. After transfection for 72 h, the cells were observed by inverted fluorescence microscopy. The results showed no green fluorescence in the blank control group without transfection, and the negative control group and the miR-183 silenced expression groups showed green fluorescence with a transfection efficiency of more than 80% (Figure 4A-F). Subculturing of the cells of interest was continued, and qPCR confirmed that miR-183 expression was downregulated in H1299R cells (Figure 4G). H1299R-shRNA-miR183 stably expressed miR-183, and H1299R-shRNA-NC cells were used as a negative control.

**Figure 5:**
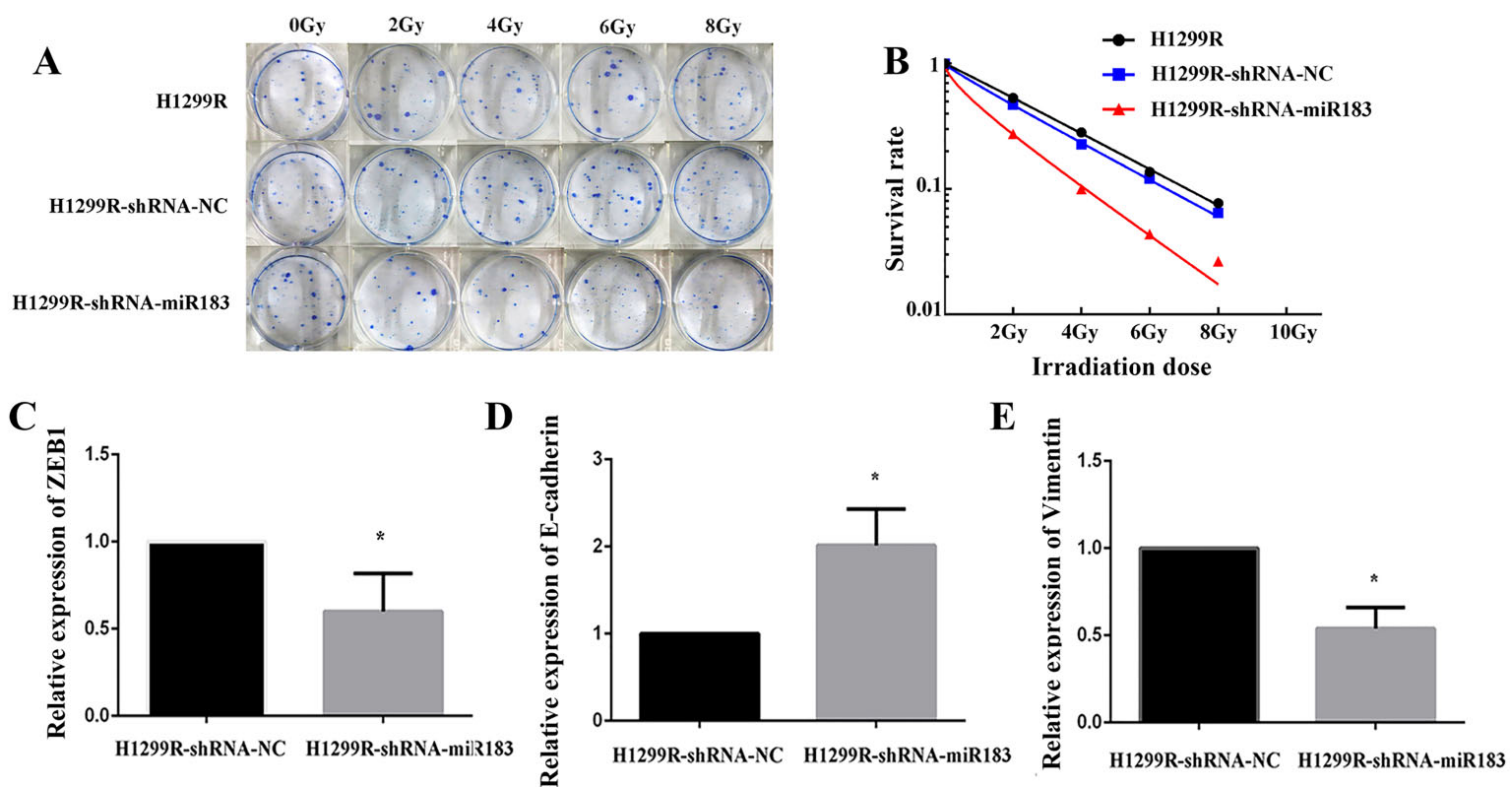
**A**, Colony formation result map (observation of >50 cell colonies under the microscope to indicate effective cell colonies). **B**, The cell survival curve under different doses was fitted by a multitarget model. **C**-**E**, mRNA expression of ZEB1, E-cadherin, and vimentin in the H1299R negative control (NC) group and the H1299R miR-183-downregulated expression group. Data are reported as means±SD. *P<0.05 (*t*-test).

After miR-183 was downregulated, cell proliferation experiments were performed. The results showed that the H1299R miR-183-downregulated expression group demonstrated a significant decrease on days 4-7 compared with the H1299R negative control group (P<0.05) (Figure 4H). These results indicated that H1299R cells have increased sensitivity to X-rays and decreased resistance after downregulation of the expression of miR-183. In the cell migration experiment, we found that scratch healing was faster in the H1299R cells and H1299R negative control cells than in the H1299R miR-183-downregulated cells (Figure 4I). These results suggested that the expression of miR-183 was downregulated and that the migratory ability of H1299R cells was weakened. The colony formation results showed that the number of colonies formed in the miR-183-downregulated group was lower than that in the blank control and negative control groups, the colony formation ability of the miR-183-downregulated group was weakened, and radiation tolerance was decreased (Figure 5A). The multitarget single-hit model was used to fit the cell survival scores of the cells with different measurements, and then, the radiosensitivity parameters of each group were calculated. The results showed that the D1, Dq, and SF2 values of the H1299R miR-183-downregulated group were significantly lower than the H1299R blank control and H1299R negative control groups (Figure 5B and Table 3), indicating that after the downregulation of miR-183 in H1299R cells, the colony-forming ability was decreased, and the radiosensitivity parameter analysis demonstrated that the cell survival shoulder region was narrowed, and the radiological resistance was weakened.

Next, qPCR was performed to detect the differences in the expression of ZEB1, E-cadherin, and vimentin at the mRNA level. The results showed that in H1299R cells, after downregulating the expression of miR-183, the mRNA expression levels of ZEB1 and vimentin were decreased, and the mRNA expression level of E-cadherin was significantly increased (P<0.05) (Figure 5C-E). Western blotting showed that after downregulating the expression of miR-183 in H1299R cells, the protein expression levels of ZEB1 and vimentin were significantly decreased compared with those in the negative control cells (P<0.05), while the E-cadherin protein expression level showed a significantly increasing trend (P<0.05) (Figure 2A and C).

**Figure 6:**
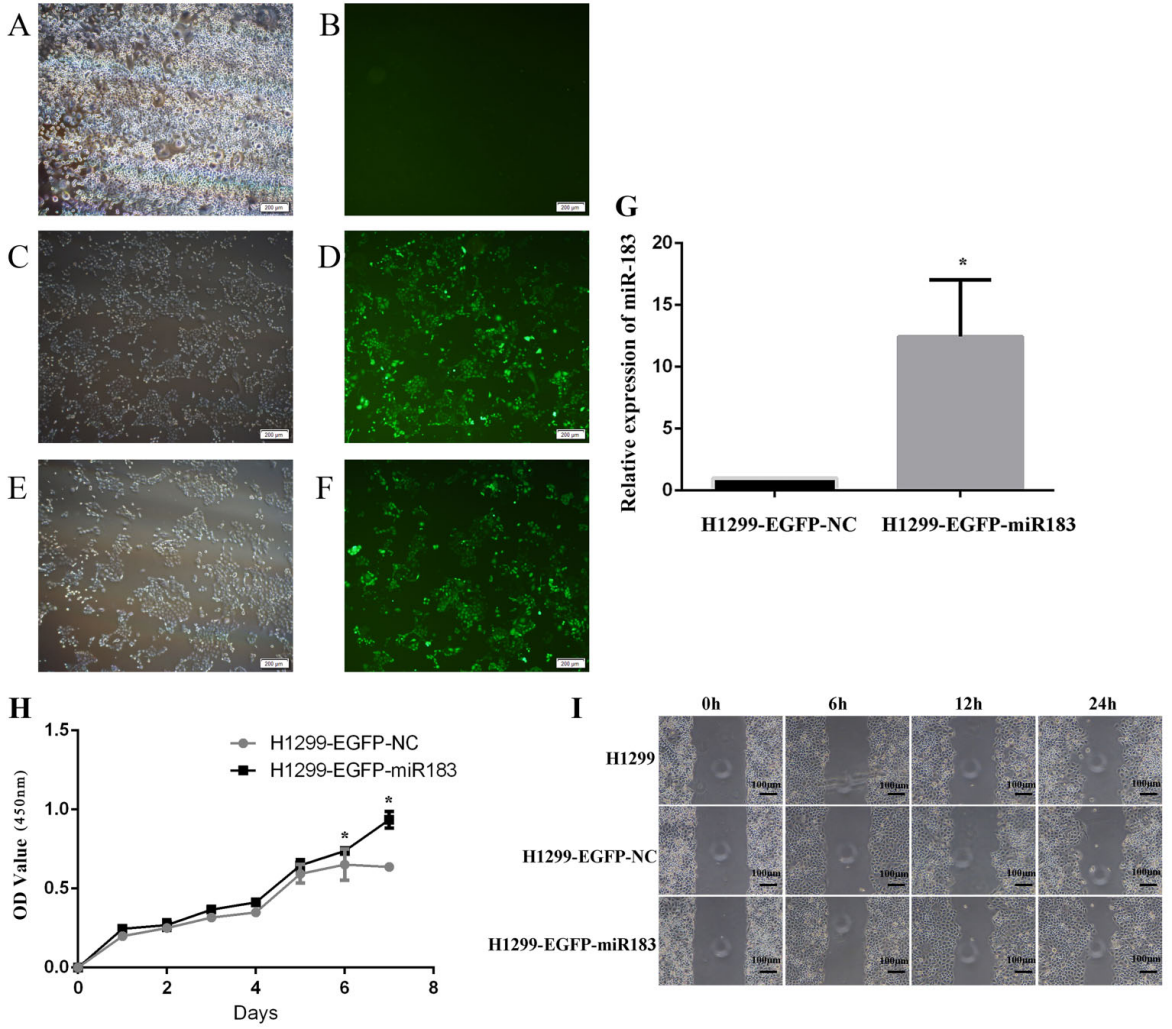
**A-F**, The expression of EGFP under a fluorescence microscope after H1299 cells were infected with lentivirus (**A** and **B**, H1299 blank control group; **C** and **D**, H1299 negative control group; **E** and **F**, H1299 miR-183-overexpression group) (scale bar: 200 μm). **G**, The expression of miR-183 was detected by qPCR after transfection with lentivirus in H1299 cells. **H**, The effect on cell proliferation after H1299 cells were infected with virus (H1299-EGFP-NC: H1299 negative control group; H1299-EGFP-miR183: H1299 miR-183-upregulated expression group). Data are reported as means±SD (n=3). *P<0.05 (*t*-test). **I**, Scratch healing test after H1299 cells were infected with virus.

Furthermore, a lentiviral transfection assay was performed to upregulate the expression of miR-183 (Figure 6A-F). qPCR experiments were performed to verify the overexpression of miR-183 in H1299 cells (P<0.05) (Figure 6G). The results showed that the H1299-EGFP-miR-183 cell overexpressed miR-183.

**Figure 7:**
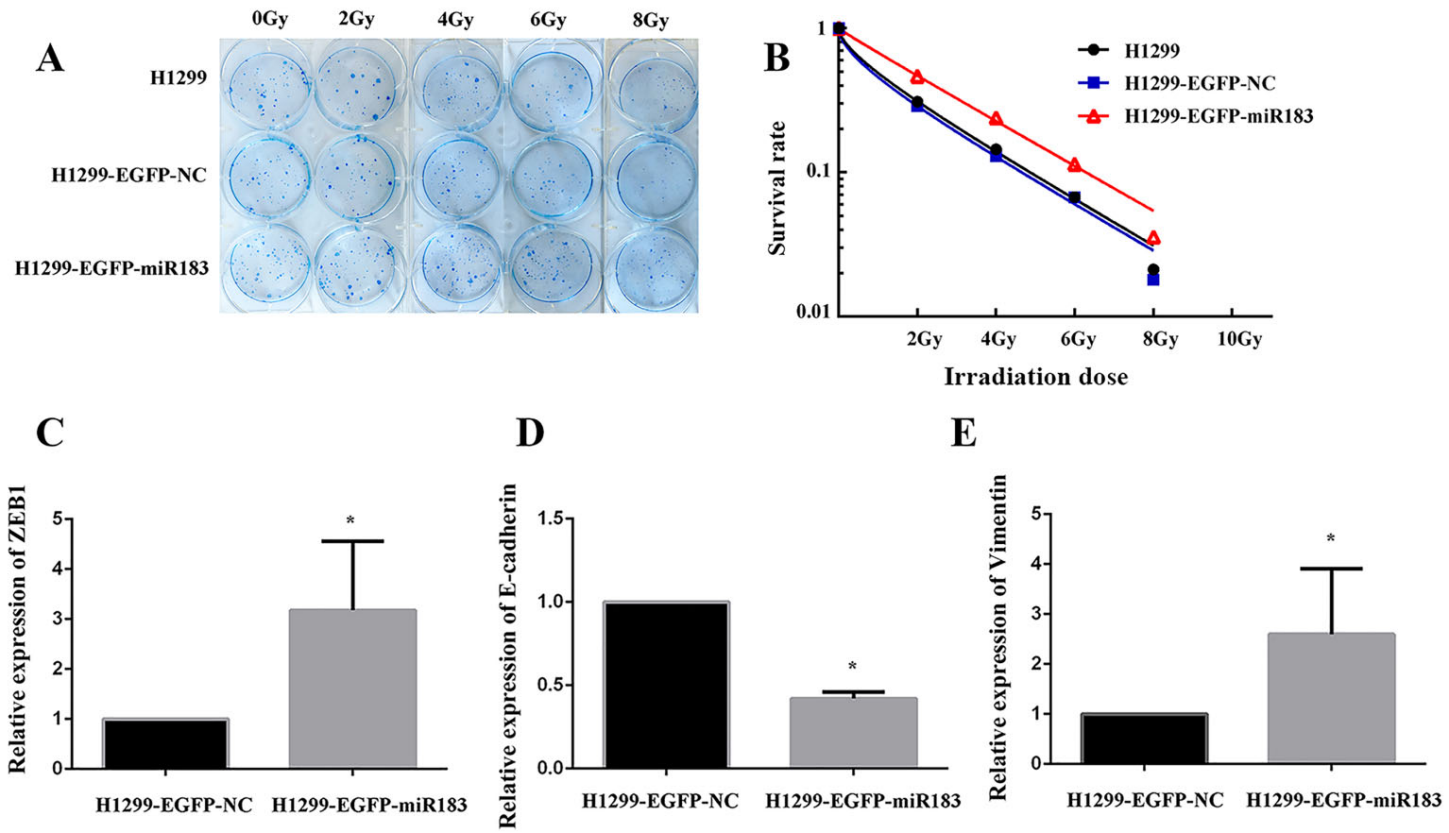
**A**, Colony formation result map (observation of >50 cell colonies under the microscope to indicate effective cell colonies). **B**, The cell survival curve under different doses was fitted by a multitarget model. **C**-**E**, The mRNA expression of ZEB1, E-cadherin, and vimentin mRNA in the H1299 negative control group and H1299 miR-183-overexpression group. Data are reported as means±SD (n=3). *P<0.05 (*t*-test).

After miR-183 was upregulated, the results of the cell proliferation assay showed that the proliferation of the H1299 miR-183-upregulated cells was significantly higher than that of the negative control cells on days 5-7 (P<0.05) (Figure 6H). This result indicated that H1299 cells had decreased sensitivity to X-rays and increased resistance after the expression of miR-183 was upregulated. The results of the cell migration assay showed that the H1299 parental cell group and H1299 negative control group demonstrated a faster scratch healing rate than H1299 miR-183-upregulated expression group in the same period of time (Figure 6I). The results of the colony formation experiments showed that at the same irradiation dose, the number of colonies formed in the miR-183-upregulated group was increased and the clonality and radiation tolerance were enhanced (Figure 7A). A multitarget model to fit the different measurements of the cell survival scores was used to calculate the radiosensitivity parameters of each group. The results showed that D0, Dq, and SF2 were increased in the H1299 upregulated group compared with the H1299 blank control group and the H1299 negative control group (Figure 7B and Table 3). These results indicated that when the expression of miR-183 was upregulated in the cells, their colony-forming ability and radioresistance were enhanced.

qPCR was used to detect mRNA expression, and the ZEB1 and vimentin mRNA expression levels were increased, while the E-cadherin mRNA expression levels were decreased in the H1299 miR-183-upregulated group (P<0.05) (Figure 7C, D, and E). Western blotting detected the effect of the upregulated expression of miR-183 on the protein expression of ZEB1, E-cadherin, and vimentin. The results showed that the protein expression levels of ZEB1 and vimentin were significantly increased while the protein expression level of E-cadherin was significantly decreased in the upregulated miR-183 group compared with the negative control group (Figure 3A and D).

## Discussion

Radiotherapy is still one of the main treatment strategies for lung cancer, but due to radiotherapy resistance, its use has shown reduced efficacy. Recent studies have shown that radiation exposure can induce EMT in tumor cells and enhance the invasion and migratory abilities of tumor cells. In the early stage of our research, morphological changes were observed when radioresistance was induced in H1299R cells, which in turn transformed epithelial cells into mesenchymal cells. In addition, miR-183 was highly expressed in H1299R cells. Informatics analysis revealed that miR-183 can directly regulate the expression of ZEB1. In this experiment, we detected the expression of ZEB1 and EMT-related markers in H1299 and H1299R cells by qPCR and western blotting. The results revealed that the expression of ZEB1 and vimentin was increased, and the protein expression levels of E-cadherin were decreased in H1299R cells. These findings strongly confirmed that EMT occurs in radioresistant lung adenocarcinoma cells.

In this study, downregulating the expression of miR-183 in H1299R cells weakened the radioresistance of H1299R cells and decreased their proliferation and migratory abilities, which might in turn reverse the process of EMT. Conversely, when the expression of miR-183 was upregulated in H1299 cells, the results showed that the radioresistance of H1299 cells was increased, and the proliferation and migratory abilities were enhanced. These results showed that overexpression of miR-183 might promote the process of EMT.

MiR-183 is an important member of the miRNA family and is abnormally expressed in various tumor tissues. Compared with normal tissues, different tumors express different levels of miR-183 (12). Most studies have suggested that miR-183 can be overexpressed as an oncogene in colorectal cancer (15), gastric cancer (16), breast cancer (17,18), and prostate cancer (19), and its expression might be related to tumor development and poor prognosis. However, miR-183 is expressed at low levels in osteosarcoma (20-22), promoting tumor proliferation, invasion, and metastasis.

In lung cancer studies, the relationship between the miR-183 expression levels in the blood and tissues of patients and the development, invasion, metastasis, and prognosis of lung cancer remains controversial. Some studies show that miR-183 is highly expressed in the tissues and blood of patients with lung cancer and might promote tumor formation, growth, and progression to advanced stages (23,24). Some studies suggest that miR-183 is overexpressed in lung cancer, inhibiting the invasion and metastasis of lung cancer cells (25).

In related research on the role of miR-183 in regulating the EMT process, the results are also different. In pancreatic cancer, some studies suggest that miR-183 can negatively regulate the EMT process, and reduced miR-183 expression can enhance the proliferation, migration, and invasion of pancreatic cancer cells (26). A few other studies suggest that the expression of miR-183 can promote the migration and invasion of pancreatic cancer cells, and inhibition of miR-183 expression can cause cell apoptosis (27).

In our study, we showed that miR-183 was highly expressed in H1299R cells and that this high expression mediated the EMT process to promote the occurrence of radioresistance. We not only studied the relationship between miR-183 and EMT but also explored the effects of both factors on the radiosensitivity of lung adenocarcinoma cells, and this is the first such study conducted worldwide. The findings revealed the mechanism of radioresistance and provided an experimental basis for the development of treatments to reverse radiotherapy resistance in non-small cell lung cancer. These findings bring new hope for the targeted inhibition of EMT to improve the therapeutic effect in cancer patients.

Our research has limitations, with a small amount of nonspecific detection in the WB bands. By adding other cell lines, a dual-luciferase reporter system, and mutated ZEB1 to examine the loss of the miR-183-mediate effect, we could more strongly confirm our findings.

We concluded that 1) during continuous X-ray irradiation, EMT occurred in H1299 cells, enhancing proliferative activity and increasing radioresistance; and 2) miR-183 played an important role in the process of radioresistance in H1299 cells. Downregulation of the expression of miR-183 offers hope for improving therapeutic efficacy in H1299 cells.

## Supplementary Material

Click here to view [pdf].

## References

[1] Decrock E, Hoorelbeke D, Ramadan R, Delvaeye T, De Bock M, Wang N (2017). Calcium, oxidative stress and connexin channels, a harmonious orchestra directing the response to radiotherapy treatment?. Biochim Biophys Acta Mol Cell Res.

[2] Chen GZ, Zhu HC, Dai WS, Zeng XN, Luo JH, Sun XC (2017). The mechanisms of radioresistance in esophageal squamous cell carcinoma and current strategies in radiosensitivity. J Thoracic Dis.

[3] Malik A, Sultana M, Qazi A, Qazi MH, Parveen G, Waquar S (2016). Role of natural radiosensitizers and cancer cell radioresistance: an update. Anal Cell Pathol.

[4] Baskar R, Dai J, Wenlong N, Yeo R, Yeoh KW (2014). Biological response of cancer cells to radiation treatment. Front Mol Biosci.

[5] Lee SY, Jeong EK, Ju MK, Jeon HM, Kim MY, Kim CH (2017). Induction of metastasis, cancer stem cell phenotype, and oncogenic metabolism in cancer cells by ionizing radiation. Mol Cancer.

[6] Zang C, Liu X, Li B, He Y, Jing S, He Y (2017). IL-6/STAT3/TWIST inhibition reverses ionizing radiation-induced EMT and radioresistance in esophageal squamous carcinoma. Oncotarget.

[7] Johansson AC, La Fleur L, Melissaridou S, Roberg K (2016). The relationship between EMT, CD44(high)/EGFR(low) phenotype, and treatment response in head and neck cancer cell lines. J Oral Pathol Med.

[8] Mutlu M, Raza U, Saatci O, Eyupoglu E, Yurdusev E, Sahin O (2016). miR-200c: a versatile watchdog in cancer progression, EMT, and drug resistance. J Mol Med.

[9] Yu L, Yang Y, Hou J, Zhai C, Song Y, Zhang Z (2015). MicroRNA-144 affects radiotherapy sensitivity by promoting proliferation, migration and invasion of breast cancer cells. Oncol Rep.

[10] Da C, Wu L, Liu Y, Wang R, Li R (2017). Effects of irradiation on radioresistance, HOTAIR and epithelial-mesenchymal transition/cancer stem cell marker expression in esophageal squamous cell carcinoma. Oncol Lett.

[11] Zhu C, Deng X, Wu J, Zhang J, Yang H, Fu S (2016). MicroRNA-183 promotes migration and invasion of CD133(+)/CD326(+) lung adenocarcinoma initiating cells via PTPN4 inhibition. Tumour Biol.

[12] Zhang QH, Sun HM, Zheng RZ, Li YC, Zhang Q, Cheng P (2013). Meta-analysis of microRNA-183 family expression in human cancer studies comparing cancer tissues with noncancerous tissues. Gene.

[13] Yang Li (2016). Establish a human lung adenocarcinoma resistant cell line H1299R and investigate the variation of radiosensitivity affected by interactions between HIF-1α and miR-183. [Master’s thesis].

[14] Livak KJ, Schmittgen TD (2001). Analysis of relative gene expression data using real-time quantitative PCR and the 2(-Delta Delta C(T)) method. Methods.

[15] Bi DP, Yin CH, Zhang XY, Yang NN, Xu JY (2016). MiR-183 functions as an oncogene by targeting ABCA1 in colon cancer. Oncol Rep.

[16] Cao LL, Xie JW, Lin Y, Zheng CH, Li P, Wang JB (2014). miR-183 inhibits invasion of gastric cancer by targeting Ezrin. Int J Clin Exp Pathol.

[17] Chang CW, Wu HC, Terry MB, Santella RM (2015). microRNA expression in prospectively collected blood as a potential biomarker of breast cancer risk in the BCFR. Anticancer Res.

[18] Cheng Y, Xiang G, Meng Y, Dong R (2016). MiRNA-183-5p promotes cell proliferation and inhibits apoptosis in human breast cancer by targeting the PDCD4. Reprod Biol.

[19] Ueno K, Hirata H, Shahryari V, Deng G, Tanaka Y, Tabatabai ZL (2013). microRNA-183 is an oncogene targeting Dkk-3 and SMAD4 in prostate cancer. Br J Cancer.

[20] Mu Y, Zhang H, Che L, Li K (2014). Clinical significance of microRNA-183/Ezrin axis in judging the prognosis of patients with osteosarcoma. Med Oncol.

[21] Zhao H, Guo M, Zhao G, Ma Q, Ma B, Qiu X (2012). miR-183 inhibits the metastasis of osteosarcoma via downregulation of the expression of Ezrin in F5M2 cells. Int J Mol Med.

[22] Zhu J, Feng Y, Ke Z, Yang Z, Zhou J, Huang X (2012). Down-regulation of miR-183 promotes migration and invasion of osteosarcoma by targeting Ezrin. Am J Pathol.

[23] Wang J, Li Z, Ge Q, Wu W, Zhu Q, Luo J (2015). Characterization of microRNA transcriptome in tumor, adjacent, and normal tissues of lung squamous cell carcinoma. J Thoracic Cardiovasc Surg.

[24] Zhu W, Zhou K, Zha Y, Chen D, He J, Ma H (2016). Diagnostic value of serum miR-182, miR-183, miR-210, and miR-126 levels in patients with early-stage non-small cell lung cancer. PloS One.

[25] Wang G, Mao W, Zheng S (2008). MicroRNA-183 regulates Ezrin expression in lung cancer cells. FEBS Lett.

[26] Lin X, Zheng L, Song H, Xiao J, Pan B, Chen H (2017). Effects of microRNA-183 on epithelial-mesenchymal transition, proliferation, migration, invasion and apoptosis in human pancreatic cancer SW1900 cells by targeting MTA1. Exp Mol Pathol.

[27] Lu YY, Zheng JY, Liu J, Huang CL, Zhang W, Zeng Y (2015). miR-183 induces cell proliferation, migration, and invasion by regulating PDCD4 expression in the SW1990 pancreatic cancer cell line. Biomed Pharmacother.

